# Integrated analysis of transcriptomics, proteomics and metabolomics data reveals the role of SLC39A1 in renal cell carcinoma

**DOI:** 10.3389/fcell.2022.977960

**Published:** 2022-11-03

**Authors:** Yulin Yuan, Zimeng Liu, Bohan Li, Zheng Gong, Chiyuan Piao, Yang Du, Bo Zhan, Zhe Zhang, Xiao Dong

**Affiliations:** ^1^ Department of Urology, The First Hospital of China Medical University, Shenyang, Liaoning, China; ^2^ Department of Anesthesiology, The First Hospital of China Medical University, Shenyang, Liaoning, China

**Keywords:** metabolic reprogramming, multi-omics analysis, SLC39A1, renal cell carcinoma, signal transduction

## Abstract

**Purpose:** Accumulating evidence suggests that solute carrier family 39 member 1 (SLC39A1) conceivably function as a tumor suppressor, but the underlying mechanism in renal cell carcinoma (RCC) is poorly understood.

**Methods:** OSRC-2 renal cancer cells were first transfected with SLC39A1 overexpressed vectors and empty vectors and then used in transcriptomics, proteomics, and metabolomics integrated analyses.

**Results:** SLC39A1 significantly altered several metabolisms at transcriptional, protein and metabolic levels, including purine and pyrimidine metabolism, amino acids and derivatives metabolism, lactose metabolism, and free fatty acid metabolism. Additionally, SLC39A1 could promote ferroptosis, and triggered significant crosstalk in PI3K-AKT signal pathway, cAMP signal pathway, and peroxisome proliferators-activated receptor (PPAR) signal pathway.

**Conclusion:** We found SLC39A1 transfection impaired tumor metabolism and perturbed tumor metabolism-related pathways, which was a likely cause of the alteration in cell proliferation, migration, and cell cycle progression in RCC cells. These multi-omics analyses results provided both a macroscopic picture of molecular perturbation by SLC39A1 and novel insights into RCC tumorigenesis and development.

## 1 Introduction

Renal cell carcinoma (RCC), the most universal renal malignancies, ranked 8th in all estimated cancer cases in 2021. The past few decades have witnessed steadily growing incidence and mortality of RCC in worldwide (Siegel et al., 2021). The new cases and new deaths of RCC on a global scale were 431,288 and 179,368 in 2020 (Sung et al., 2021). The blurred knowledge of oncogenic molecular mechanism and the insensitivity to chemoradiotherapy after metastasis contribute to inefficient clinical management of RCC patients (Lalani et al., 2019). Hence, there has been growing interest in understanding RCC pathogenesis to enhance diagnostic sensitivity and improve therapeutic outcomes.

Recently, increasing evidence indicated the relation between tumorigenesis and intricate changes in metabolic pathways, which was so-called metabolic reprogramming (Wettersten et al., 2017). The Warburg Effect, an emerging hallmark of tumor metabolism reprogramming, referred to the phenomenon that tumor cells were prone to increasing glucose absorption and facilitating its conversion to lactate regardless of normal mitochondria and sufficient oxygen. The glycolytic switch in cancer cells allowed glycolytic intermediates metabolites to participate in nucleotide and amino acid synthesis *in vivo* and therefore, sustained long-term tumor growth, proliferation, and survival (Hanahan and Weinberg, 2011; Liberti and Locasale, 2016). A few studies had posed that RCC was a metabolic disease. The development of RCC was characterized by the alteration in glucose metabolism and tricarboxylic acid cycle (TCA cycle). ATP and other organic molecules produced in reprogrammed carbohydrate metabolic processes, such as upregulated aerobic glycolysis and pentose phosphate pathway, enabled renal cancer cells to tolerate various stress and immune damage (Chakraborty et al., 2021). And lipid synthesis was reported to be downregulated and significant alteration in fatty acid (FA) metabolism was discovered in RCC. Several FA metabolic enzymes had been identified as potential clinical prognostic markers (Zhao et al., 2019; Chakraborty et al., 2021). Moreover, amino acid metabolism was also reprogrammed. Arginine and tryptophan levels were reduced in RCC (Wise and Thompson, 2010). The glutamine metabolism of renal cancer cells had long been studied. Glutaminolysis generated amount of α-ketoglutarate to maintain TCA cycle and provides nitrogen for protein and nucleotide synthesis (Weiss, 2018). It was also speculated that elevated glutamine utilization facilitates RCC cells to attenuate oxidative stress by feeding the glutathione/glutathione disulfide (GSH/GSSG) antioxidant system, rather than for energy production in TCA cycle, thereby obtaining survival advantages (Wettersten et al., 2015). Notably, nucleotide metabolism had received little attention, which needed further research.

SLC39A1, also known as ZIP1, was responsible for transferring zinc ions into cells. There are 14 protein members in ZIP family. There were 8 transmembrane domains in ZIP proteins and zinc transport was thought to be related to an intracellular loop located between domains III and IV (Guerinot, 2000; Milon et al., 2006). Native and endogenous ZIP1 showed intracellular distribution and partly resided in endoplasmic reticulum (ER) in epithelial cells and prostate cancer cells, but in erythroid cells ZIP1 was sorted to the plasma membrane (Milon et al., 2001). SLC39A1 was reduced and inhibited tumor progression in prostate cancer (Golovine et al., 2008). In ovarian, colon, stomach, and lung mucinous carcinomas, SLC39A1 also showed significant and persistent low expression (Desouki et al., 2015). Our previous work had confirmed SLC39A1 demonstrated low expression in RCC tissues and the expression of SLC39A1 was negatively correlated with Fuhrman stage and clinical stage. Knocking down SLC39A1 remarkably promoted tumor proliferation and invasion ability and miR-223 was responsible for the dysregulation of SLC39A1 in RCC (Dong et al., 2014; Dong et al., 2018). Zinc was an essential microelement that was gaining momentum for its anti-cancer activity in multiple cancers (Hoang et al., 2016). Zinc took part in lipid and glucose metabolism and impaired cellular energy production and triggered accumulation of reactive oxygen species (ROS) *via* inhibiting glycolysis and the mitochondrial electron transport chain (Dineley et al., 2003; Olechnowicz et al., 2018a). Besides, zinc altered choline metabolism, leading to lymphoma cell apoptosis (Yoon et al., 2021). Thus, we hypothesized the altered landscape of metabolism and signal transduction pathways caused by SLC39A1 might lie behind its inhibition effect on tumorigenesis and malignant progression in RCC.

In our work, transcriptomics, proteomics, and metabolomics studies were performed on human renal cancer cell (OSRC-2) samples, composed of cells transfected with the negative control virus (NC) and SLC39A1 lentivirus (ZIP), aiming to identify key alteration arising from SLC39A1 in RCC. Transcriptomics analysis was applied to uncover the alteration in genes and proteomics analysis indicated functional changes. Metabolomics reflected specific metabolic changes. Finally, we integrated three sets of data to figure out significantly perturbed pathways at transcription, protein and metabolism levels and to identify potential downstream molecules for SLC39A1 that may aid in understanding its tumor suppression effects, thereby providing new insights for RCC diagnosis and treatment.

## 2 Materials and methods

### 2.1 Cell culture

OSRC-2 cells were obtained from Type Culture Collection Cell Bank of Chinese Academy of Sciences (Shanghai, China). RPMI-1640 medium (HyClone, Logan, UT, United States) supplemented with 10% fetal bovine serum (FBS; Biological Industries, Beit-HaEmek, Israel) was used to continuously culture OSRC-2 cells. OSRC-2 cells were cultured in a incubator with suitable humidity under normoxia (5% CO_2_ and 95% humidity).

### 2.2 Cell transfection

The transfection of overexpression plasmid with empty and SLC39A1 vetors (GeneChem, Shanghai, China) was strictly followed the manufacturer’s protocols. The accession number of SLC39A1 was NM_014437. The transfected cells were harvested for subsequent assays after selected with puromycin (2 µg/ml) over 72 h.

### 2.3 Western blotting assay

The application of antibodies to SLC39A1 (sc-393345, Santa Cruz Biotechnology) and ACTB (4970S, Cell Signaling Technology) were under the manufacturers’ instruction. Western blotting assays was carried out as previously mentioned (Dong et al., 2018). In brief, the lysates (RIPA buffer: PMSF = 100:1) were used to extract total proteins. 50 µg of standardized proteins was subjected to SDS-PAGE (10%) in electrophoresis and then transferred onto PVDF membranes (Bio-Rad, SA). The blocking solution, TBS-T with 5% (w/v) skim milk powder, was used to block non-specific binding. Then the membranes were applied with incubated with primary and secondary antibodies. The ECL reagents (TransGen Biotechnology, Beijing, China) on a DNR Bio-Imaging Systems (Mahale HaHamisha, Israel) was used to visualize immunoblot bands. The ImageJ 1.46r software (Wayne Rasband, National institutes of Health, Bethesda, MA, United States) was used to calculate densitometric values. The tatistical analysis were performed on the ratios of SLC39A1/ACTB.

### 2.4 RNA extraction and quantitative real-time PCR assay

The RNAiso Plus was used to total RNA. The PrimeScript™ RT Master Mix was used to synthesize cDNA. qRT-PCR was then performed using SYBR^®^ Pre-mix Ex TaqTM (Tli RNaseH Plus) on LightCyclerTM 480II system (Roche, Basel, Switzerland). Reagents above were purchased from Takara Biotechnology (Dalian, China). The primer sequences for SLC39A1 were as follows: F: 5′-GCT​GTT​GCA​GAG​CCA​CCT​TA-3’; R: 5′-CAT​GCC​CTC​TAG​CAC​AGA​CTG-3’. The primer sequences for ACTB were as follows: F: 5′-CAT​GTA​CGT​TGC​TAT​CCA​GGC-3’; R: 5′-CTC​CTT​AAT​GTC​ACG​CAC​GAT-3’. The -ΔΔCT method was applied to quantify the relative genes expression in three independent experiments.

### 2.5 Transcriptomics analysis

#### 2.5.1 RNA Extraction and RNA detection

3 pairs of samples were used for both transcriptomic and proteomics analysis, transfected with empty vectors (NC) and SLC39A1 overexpressed vector (ZIP), respectively. The extraction of RNA was conducted as described above. The integrity of RNA and the possibility of DNA contamination in RNA samples were analyzed by agarose gel electrophoresis, Qubit 2.0 fluorometer and Agilent 2100 bioanalyzer.

#### 2.5.2 Library construction

Two approaches were taken to obtain mRNAs: Oligo (dT) magnetic beads were used to enrich the mRNAs with poly-A tails; Ribosomal RNAs were removed from total RNA to purify mRNAs. mRNAs were broken into short fragments by fragmentation buffer. Short fragment RNA was served as templates and the first strand cDNA was synthesized from corresponding template RNA with Random Hexanucleotide Primers. And two-strand cDNAs were synthesized with the presence of buffer, dNTPs, and DNA polymerase I. AMPure XP beads are used to purify the double-strand cDNAs. Then the purified double-stranded cDNAs underwent the repair of cDNA ends, the A-tail addition, and the ligation of sequencing adapter. cDNA fragments with compatible length were selected by AMPure XP beads and then enriched by PCR to obtain the final cDNA library. The quality of cDNA library was tested by following methods: Qubit2.0 was supplied for preliminary quantification. Agilent 2100 was used to detect insert size of the library in line with expectation. The effective concentration of the library was accurately quantified through Q-PCR according to the quality control metrics: the effective concentration of library >2 nM.

#### 2.5.3 Basic analysis

cDNA libraries were sequenced with the Illumina HiSeq high-throughput sequencing platform to get image files, which were transformed by CASAVA base identification to obtain raw data in FASTQ format. Cutadapt (v1.15) software was used to filter the sequencing data. The filtered data was qualitified following QC metrics: 1) The sequencing error rate was represented by e, and base quality value in Illumina was represented by Qphred, Qphred = −10log10(e). The sequencing error rates of samples was controlled below the standard (0.05%); 2) the GC content distribution of samples was controlled at around 50%. The qualified data were mapping to the reference genome using HISAT2 (http://ccb.jhu.edu/software/hisat2/index.shtml) and the default mismatch was controlled below 2. Then the alignment region distribution of mapped reads was calculated.

#### 2.5.4 mRNA analysis

First, HTSeq (0.9.1) statistics was used to compare the Read Count values on each gene as the original expression of the gene, and then FPKM was applied for standardization of gene expression. Then differentially expressed genes (DEGs) were screened by DESeq (1.30.0) with standards as follows: the expression difference multiple |log2FoldChange| > 1 and significant *p*-value < 0.05. R language Pheatmap (1.0.8) software package was used to perform bi-directional clustering analysis of all different genes of samples. The volcano map was obtained according to the expression level of the same gene in different samples and the expression patterns of different genes in the same sample with Euclidean method to calculate the distance and Complete Linkage method to cluster. Next, all the genes were mapped to Kyoto Encyclopedia of Genes and Genomes, the Gene Ontology and Reactome database to annotate metabolic pathways and signaling pathways that DEGs mainly participated in. Finally, Disease ontology (DO) and DisGeNET databases were used to uncover human diseases with DEGs altered.

### 2.6 Proteomics analysis

#### 2.6.1 Sample preparation

Cell samples were mixed with reaction solution and sonicated for 10 min in ice-water bath and the reaction was performed at 60 °C for 30 min. Protein concentration was measured by Bradford method. Trypsin was added at a ratio of 1:50 (enzyme: protein, w/w) for overnight digestion at 37 °C. After centrifugation (12,000×g, 15 min), the supernatant was subjected to peptide purification using self-made desalting columns and stored at −20 C for later use. The peptides were reconstituted in TMT reagent buffer, and the samples were separately labeled with different TMT labeling reagents. The labeled samples were then mixed and subjected to Sep-Pak C18 desalting and were fractionated using high pH reverse phase chromatography and combined into 15 fractions. Media samples were filtered through 0.22 µm filters. Each filtered media was concentrated to a final volume of 200 µL using 10 kDa AMICON Ultra-15 Centrifugal Filters. Concentrated media were mixed with equal volume of 2X reaction solution (2% SDC, 20 mM TCEP, 80 mM CAA).

#### 2.6.2 LC-MS/MS analysis

LC-MS/MS data acquisition was carried out on a Q Exactive plus mass spectrometer coupled with an Easy-nLC 1200 system (both Thermo Scientific). Peptides were first loaded onto a C18 trap column (75 μm × 2 cm, 3 µm particle size, 100 Å pore size, Thermo) and then separated in a C18 analytical column (75 μm × 250 mm, 2 µm particle size, 100 Å pore size, Thermo). Mobile phase A (0.1% formic acid) and mobile phase B (80% ACN, 0.1% formic acid) were used to establish the separation gradient. A constant flow rate was set at 300 nL/min. For DDA mode analysis of TMT samples, each scan cycle is consisted of one full-scan mass spectrum (R = 70 K, AGC = 3e6, max IT = 50 ms, scan range = 350–1800 m/z) followed by 15 MS/MS events (R = 35 K, AGC = 1e5, max IT = 50 ms). HCD collision energy was set to 32. Isolation window for precursor selection was set to 1.2 Da. Former target ion exclusion was set for 45 s.

#### 2.6.3 data Research and data analysis

MS/MS spectrum of the cell samples were analyzed with MaxQuant (v1.6.6) using the Andromeda database search algorithm (Tyanova, Temu, and Cox 2016). The MS1 match tolerance was set as 20 ppm for the first search and 4.5 ppm for the main search; the MS2 tolerance was set as 20 ppm. Then the spectrum was filtered through Uniprot database search to obtain peptides with more than 99% confidence and then the false discovery rate FDR verification is performed. Peptides and proteins with FDR greater than 1% were removed. Quality controls were performed with criteria as follows: peptide length distribution of 7-40 amino acids, missed cleavage sites = 0, measurement error = ± 10 ppm. The identified protein were annotated from the following database: GO, KEGG and COG annotation were performed by BLAST against the GO, KEGG and COG databases (blast, e-value ≤ 1e−5), using BLAST and Uniprot, InterPro database for domain annotation. Further analysis was performed using the “proteingroups.txt” file produced by MaxQuant. The reporter intensities were calculated. A student’s t-tests were performed comparing the groups. Fold change and *p*-values of the proteins were calculated. Proteins with a *p*-value <0.05 and fold change >1.5 were identified as significant changed proteins between groups.

### 2.7 Metabolomics analysis

#### 2.7.1 Sample preparation and extraction

6 pairs of Cell samples subjected to metabolomics analysis for more reliable and significant difference in metabolites and metabolic pathways between NC and ZIP groups. Samples were thawed on ice and added with 1 ml 80% methanol aqueous solution, then whirled for 2 min. The mixture was frozen for 3 min in liquid nitrogen and then whirled for 2 min (repeat 3 times). Then the mixture was centrifuged at 12,000 r/min for 10 min at 4 C. Finally, 200 μL of supernatant was taken into the inner liner of the corresponding injection bottle for on-board analysis.

#### 2.7.2 T3 UPLC

The sample extracts were analyzed by using LC-ESI-MS/MS system (UPLC, ExionLC AD; MS, QTRAP^®^ System). The analytical conditions were as follows: UPLC column, Waters ACQUITY UPLC HSS T3 C18 (1.8μm, 2.1 mm*100 mm); column temperature, 40 C; flow rate, 0.4 ml/min; injection volume, 2 μL or 5μL; solvent system, water (0.1% formic acid): acetonitrile (0.1% formic acid); gradient program, 95:5 V/V at 0 min, 10:90 V/V at 10.0 min, 10:90 V/V at 11.0 min, 95:5 V/V at 11.1 min, 95:5 V/V at 14.0 min.

#### 2.7.3 QTOF-MS/MS

The Triple TOF mass spectrometer was applied for acquiring MS/MS spectra on an information-dependent basis (IDA) during an LC/MS experiment. The acquisition software (TripleTOF 6600, AB SCIEX) collects and triggers the acquisition of MS/MS spectra depending on pre-selected criteria. In each cycle, 12 precursor ions with intensity greater than 100 were chosen for fragmentation at collision energy (CE) of 30 V (12 MS/MS events with product ion accumulation time of 50 msec each). ESI source conditions were set as follows: Ion source gas 1 as 50 Psi, Ion source gas 2 as 50 Psi, Curtain gas as 25 Psi, source temperature 500°C, Ion Spray Voltage Floating (ISVF) 5500 V or −4500 V in positive or negative modes, respectively.

#### 2.7.4 ESI-Q TRAP-MS/MS

LIT and triple quadrupole (QQQ) scans were acquired on a triple quadrupole-linear ion trap mass spectrometer (QTRAP). QTRAP^®^ LC-MS/MS System was equipped with an ESI Turbo Ion-Spray interface, operating in positive and negative ion mode, and controlled by Analyst 1.6.3 software (Sciex). The ESI source operation parameters were as follows: source temperature, 500°C; ion spray voltage (IS) 5500 V (positive), −4500 V (negative); ion source gas I (GSI), gas II (GSII), curtain gas (CUR) was set at 50, 50, and 25.0 psi, respectively; the collision gas (CAD) was high. Instrument tuning and mass calibration were performed with 10 and 100 μmol/L polypropylene glycol solutions in QQQ and LIT modes, respectively. A specific set of MRM transitions were monitored for each period according to the metabolites eluted within this period.

#### 2.7.5 Quality control for MS/MS

Quality control was carried out with following 3 criteria: 1) the total ion chromatogram (TIC map) of the sample mass spectrometry detection analysis was overlapped to judge the repeatability of metabolite extraction and detection; 2) QC samples were subjected to Pearson correlation analysis (|r | →1); 3) the proportion of substances with CV (Coefficient of Variation) value less than 0.3 in QC samples was higher than 75%.

#### 2.7.6 Qualitation and quantitation of metabolites

After LC-QTOF-MS/MS experiment, metabolites were to accurately qualitied by MWDB and Maiwei integrated public database MHK database and MetDNA. The multiple ion pair information and retention time RT (Retention time) were extracted for identifying metabolites. Simultaneously, Relative quantification of population samples was proceeded through Q- Trap performs combined with MWDB and MHK. The quantification of metabolites was conducted by the multiple reaction monitoring mode (MRM) analysis of triple quadrupole mass spectrometry. The area under the peaks of the extracted ion chromatographic peaks of all metabolites are respectively integrated, and the chromatographic peaks of the same metabolite in different samples are integrated and corrected.

#### 2.7.7 PCA, hierarchical cluster analysis and Pearson correlation coefficients

PCA (principal component analysis) was performed by statistics function prcomp within R (www.r-project.org). The data was unit variance scaled before unsupervised PCA. The HCA (hierarchical cluster analysis) results of samples and metabolites were presented as heatmaps with dendrograms, while Pearson correlation coefficients (PCC) between samples were calculated by the cor function in R and presented as only heatmaps. Both HCA and PCC were carried out by R package ComplexHeatmap. For HCA, normalized signal intensities of metabolites (unit variance scaling) are visualized as a color spectrum.

#### 2.7.8 Differential metabolites selection

Significantly regulated metabolites between groups were determined by VIP ≥ 1 and absolute Log2FC (fold change) ≥ 1. VIP values were extracted from OPLS-DA result, which was generated using R package MetaboAnalystR. The data was log transform (log2) and mean centering before OPLS-DA. To avoid overfitting, a permutation test (200 permutations) was performed.

### 2.8 Integrated analysis

Through Cytoscape 3.9.1 (https://js.cytoscape.org/), a network of genes, proteins and metabolic compounds was constructed to identify the pathways in which DEGs were significantly enriched, and to reveal the potential regulatory mechanisms between genes and metabolites. The differential metabolites and DEG expression data between NC and ZIP groups were imported into Cytoscape to comprehensively understand the gene and metabolic changes and the underlying mechanism through which SLC39A1 altered the metabolism in renal cancer cells.

### 2.9 Statistical analysis and bioinformatic analysis

Statistical analysis methods and quantitative methods for single-omics and multi-omics analysis could be found in corresponding method section, including but not limited to Student’s t-test, chi-square test, Fisher’s exact test, etc. Statistical significance *p*-values were considered <0.05 and were adjusted by the Benjamini–Hochberg FDR correction. And multiple databases were used to interpret the role of SLC39A1 in RCC. KEGG (https://www.genome.jp/kegg/; http://www.kegg.jp/kegg/compound/) was used to perform functional annotation and enrichment analysis of differential genes and differential metabolites in renal cancer cells. GO (http://www.geneontology.org), KEGG (http://www.kegg.jp/kegg/pathway.html), and COG (http://www.ncbi.nlm.nih.gov/COG/) database were used for protein function annotation. InterPro database (https://www.ebi.ac.uk/interpro/) and MultiLoc2 (http://abi.inf.uni-tuebingen.de/Services/MultiLoc2) were used for domain annotation and subcellular localization analysis, respectively. KEGG, GO and Reactome (https://reactome.org) databases revealed signal transduction pathways involved in differential genes. The DO (https://disease-ontology.org) and DisGeNET (https://www.disgenet.org) databases were used to reveal human diseases associated with differential genes. In addition, the HMDB (https://hmdb.ca) database suggested human metabolism, metabolic disease pathways, metabolite signaling, and drug activity pathways enriched by differential metabolites. MESA analysis (https://www.gsea-msigdb.org/) was performed using a metabolite database derived from MebaboAnalyst (https:// www.metaboanalyst.ca/). The datasets of known interaction proteins of SLC39A1 can be obtained at https://www.ncbi.nlm.nih.gov/gene/27173. All analyses and graphs were performed using R software (http://www.R-project.org, version 3.5.2) unless otherwise stated.

## 3 Results

### 3.1 Transcriptomics analysis results of SLC39A1-Overexpressed OSRC-2 renal cancer cells

**FIGURE 1:**
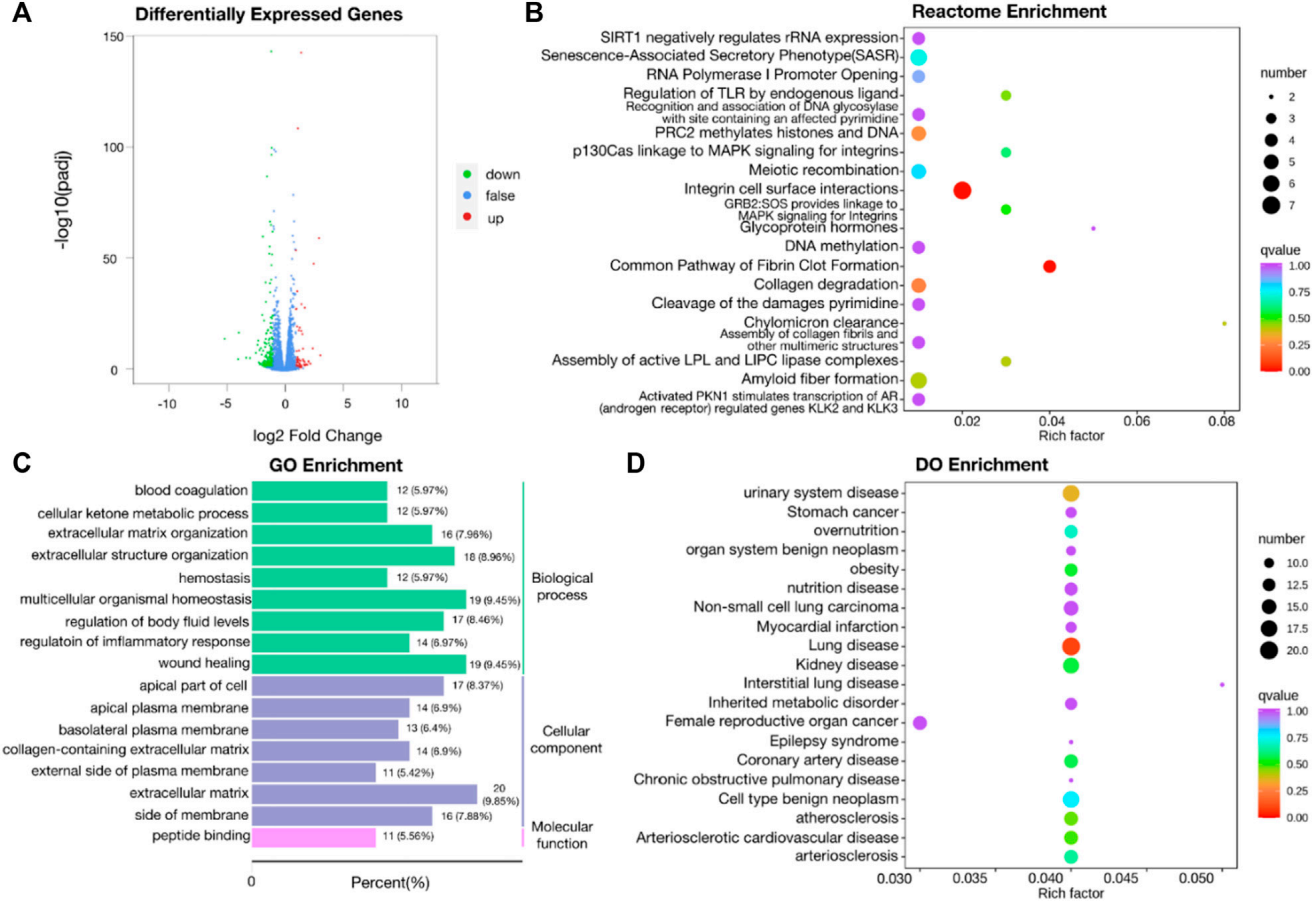
Transcriptomic analysis of SLC39A1-Overexpressed OSRC-2 cells. **(A)** The Volcano plot of differentially expressed genes. The *Y*-axis displays -log10 (p-adj) and the *X*-axis the log2 fold change of DEGs. Green dots indicate downregulated genes, and red dots indicate upregulated genes. **(B)** Gene Ontology enrichment analysis. Green bars indicate biological process (BP) category, purple bars indicate cellular component (CC) category and pink bars molecular function (MF) category. **(C)** Reactome enrichment analysis of transcriptomic data. **(D)** Disease Ontology (DO) enrichment analysis of transcriptomic data.

Two groups of OSRC-2 cells (NC and ZIP) were transfected with empty vectors and SLC39A1 vector. Then rt-PCR assays and western blot assays were conducted to test transfection efficiency (Supplementary Figures S1A,B). There were 321 DEGs following SLC39A1 overexpressed, with 71 upregulated and 250 downregulated (Figure 1A). The KEGG database was applied to interpret the specific molecular functions affected by DEGs. Altogether, 16 KEGG pathways were identified to be enriched, including nicotinate and nicotinamide metabolism, complement and coagulation cascades, neutrophil extracellular trap formation, glutathione metabolism, *Staphylococcus aureus* infection, etc. (Table 1). Subsequently, GO analysis was performed by mapping DEGs to three functional terms in GO database, and genes potentially regulated by SLC39A1 were mapped to biological processes (BP) for extracellular structure organization, multicellular organismal homeostasis, amino acid import across plasma membrane, extracellular matrix organization and wound healing; cell components (CC) for extracellular matrix, apical part of cell, basolateral plasma membrane and apical plasma membrane; molecular functions (MF) for peptide binding (Figure 1B). In addition, Reactome database integrated various reactions and biological pathways, and the result revealed prominent changes in fibrin clot formation, collagen degradation, and integrin cell surface interactions (Figure 1C). DO database was able to provide data in connection with human gene function and disease and DisGeNET database integrated genes related to human diseases. SLC39A1 triggered significant alteration in genes related to urinary system and kidney disease, lung disease, atherosclerosis, and stomach cancer (Figure 1D). Moreover, DisGeNET analysis results demonstrated remarkable changes were caused by SLC39A1 in genes associated with complement Factor I (C3 inactivator) deficiency, ischemic stroke, choriocarcinoma and hypertensive disease (Supplementary Figure S2). Besides, among the differential expressed genes, a known interaction protein of SLC39A1 was identified. Completion C5a receiver 1(C5AR1), which was significantly downregulated by SLC39A1, has been confirmed by Affinity Capture MS to directly interact with SLC39A1 (Supplementary Table S1).

**TABLE 1:** Significantly enriched pathways based on KEGG pathway analysis.

Pathway ID	Pathway Description	Up_gene	Down_gene	*p* value
ko04610	Complement and coagulation cascades	F2R; F2RL2; CFI	FGA; FGB; FGG; C5AR1	0.000218441
ko00760	Nicotinate and nicotinamide metabolism	--	NMNAT2; QPRT; NNMT; ASPDH	0.001455373
ko04613	Neutrophil extracellular trap formation	--	H2BC5; H4C8; H3C6; H2AC6;FGA;FGB;FGG;FPR1;C5AR1	0.001721488
ko00480	Glutathione metabolism	NAT8	GSTM4; GSTM3; CHAC1	0.008455026
ko05150	*Staphylococcus aureus* infection	HLA-DPA1; CFI	FGG; FPR1; C5AR1	0.01020849
ko04657	IL-17 signaling pathway	FOS; MMP3; MMP9; FOSL1	IL6	0.011662293
ko05321	Inflammatory bowel disease	HLA-DPA1	IL6; MAF; STAT4	0.012790546
ko04927	Cortisol synthesis and secretion	LDLR	CREB3L3; KCNK2; CACNA1H	0.013506465
ko04668	TNF signaling pathway	FOS; MMP3; MMP9	CREB3L3; IL6	0.023397781
ko00983	Drug metabolism - other enzymes	--	GSTM4; GSTM3; UPB1; XDH	0.027386036
ko04611	Platelet activation	COL3A1; F2R	FGA; FGB; FGG	0.032379347
ko04979	Cholesterol metabolism	LDLR	LIPC; ANGPTL4	0.034834683
ko04926	Relaxin signaling pathway	COL3A1; COL4A4; FOS; MMP9	CREB3L3	0.039795617
ko05323	Rheumatoid arthritis	FOS; HLA-DPA1; MMP3	IL6	0.040097286
ko04658	Th1 and Th2 cell differentiation	FOS; HLA-DPA1	MAF; STAT4	0.041525252
ko05322	Systemic lupus erythematosus	HLA-DPA1	H2BC5; H4C8;H3C6;H2AC6	0.044462747

### 3.2 Proteomics analysis results of SLC39A1-Overexpressed OSRC-2 renal cancer cells

**FIGURE 2:**
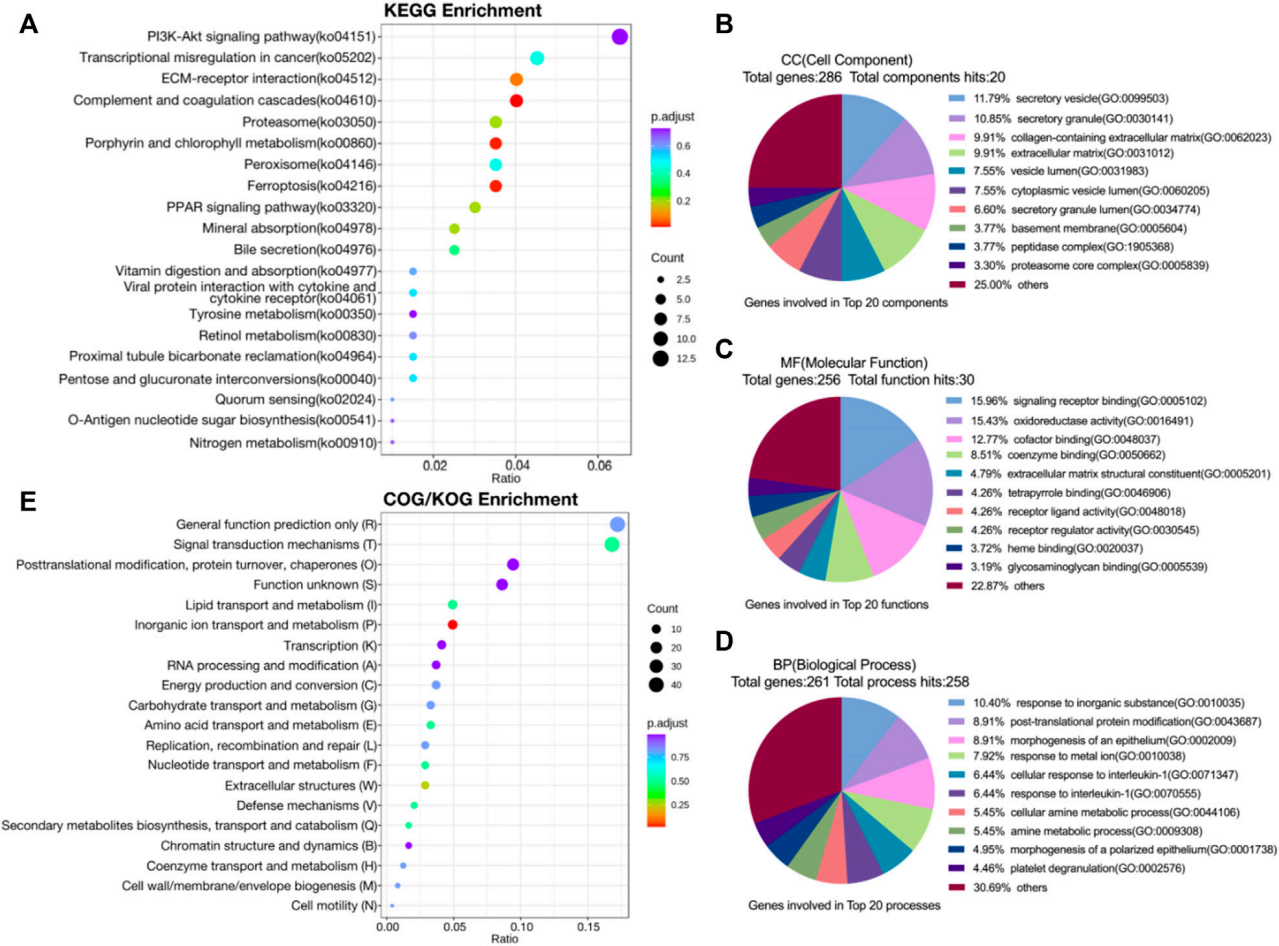
Proteomic analysis of SLC39A1-Overexpressed OSRC-2 cells. **(A)** KEGG pathway analysis of proteomic data. **(B**–**D)** Gene Ontology analysis of DEGs. **(B)** Biological Process (BP) category, **(C)** Molecular Function (MF) category and **(D)** Cellular Component (CC) category. **(E)** COG/KOG enrichment analysis of proteomic data.

The volcano map integrally demonstrated the apparent difference in protein expression levels between 2 groups, and 324 proteins were identified, with 124 upregulated and 200 downregulated (Supplementary Figure S3A). KEGG enrichment analysis revealed alteration in signal transduction pathways and metabolic process with differential proteins involved. The bubble chart indicated remarkable enrichment in PI3K-Akt signal pathway, PPAR signal pathway, complement and coagulation cascades and ferroptosis (Figure 2A). In GO enrichment analysis, differential proteins were mapped to three terms in GO database to define their main biological functions. The pie chart showed that biological processes (BP) SLC39A1 possibly regulated included but not limited to: response to inorganic substance, proteasomal ubiquitin-independent protein catabolic process, morphogenesis of an epithelium (Figure 2B). SLC39A1 also represented significant impact on molecular functions (MF) such as signaling receptor binding, oxidoreductase activity, cofactor binding, extracellular matrix structural constituent (Figure 2C). Furthermore, SLC39A1 might contribute to the formation of cell components (CC), including secretory granule and vesicle, collagen-containing extracellular matrix, and proteasome complex (Figure 2D). Cluster of Orthologous Groups of proteins (COG/KOG) enrichment analysis revealed SLC39A1 was related to nucleotide transport and metabolism, metabolism extracellular structures, and inorganic ion transport in OSRC-2 cell (Figure 2E). The discrepancy of between two groups was presumably rooted in function or localization of different domains. Subcellular localization analysis clarified specific cellular localization of differential proteins, which was closely linked to functions of proteins. Differential proteins were mainly located in cytoplasmic, nuclear and ER (Supplementary Figure S3B). And domain enrichment analysis exhibited domains of differential proteins primarily contained proteasome subunit A/B and EGF-like domain (Supplementary Figure S3C).

### 3.3 Metabolomics analysis results of SLC39A1-Overexpressed OSRC-2 renal cancer cells

**FIGURE 3:**
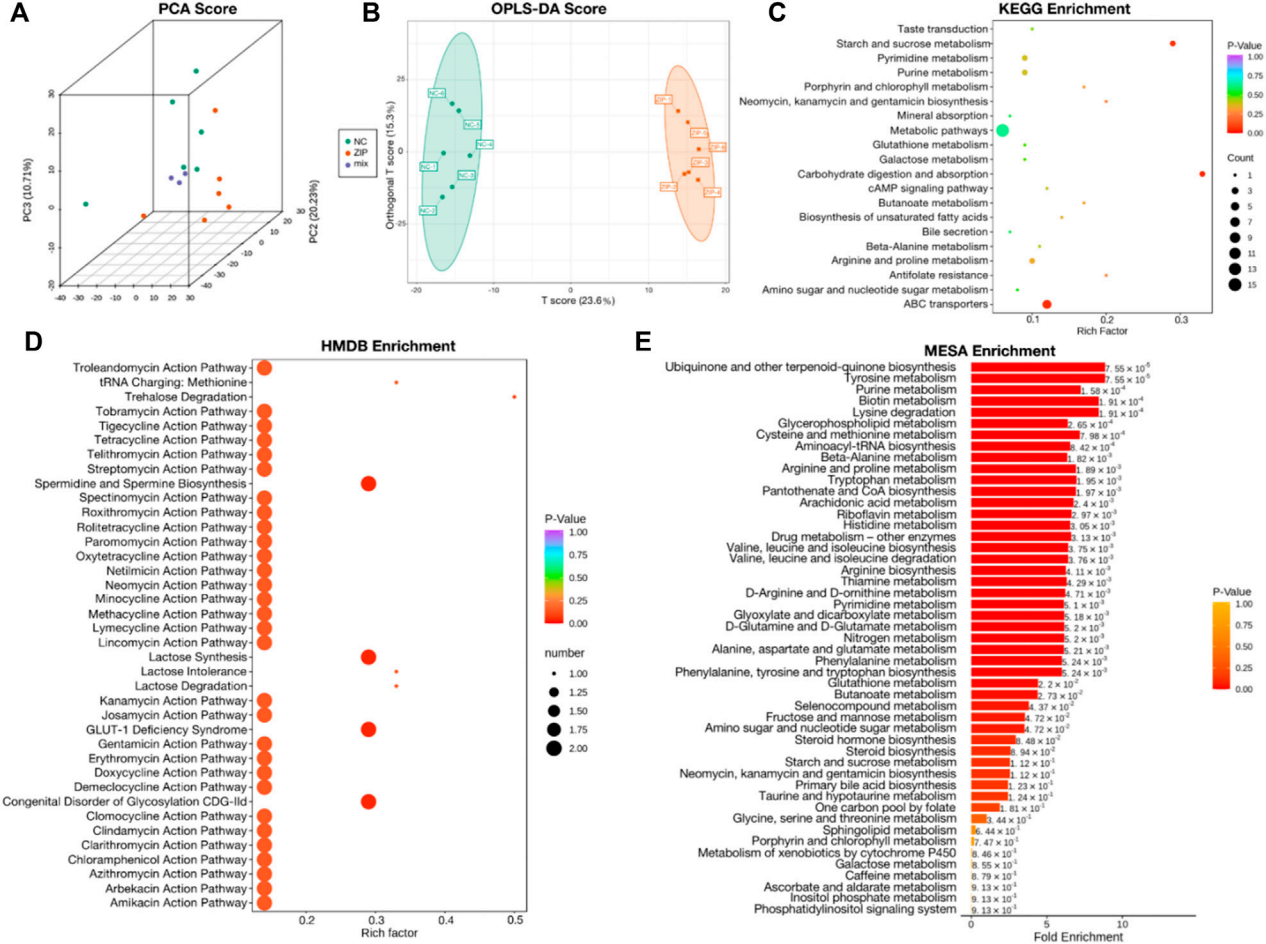
Metabolic analysis of SLC39A1-Overexpressed OSRC-2 cells. **(A)** Principal Component analysis (PCA) and **(B)** Orthogonal Partial Least Squares-Discriminant Analysis (OPLS-DA) score plots between NC and ZIP groups. **(C)** KEGG pathway analysis of metabolomic data. **(D)** HMDB enrichment of metabolic data. **(E)** MSEA enrichment of metabolic data. The label to the right of the column chart indicates *p*-value.

**FIGURE 4:**
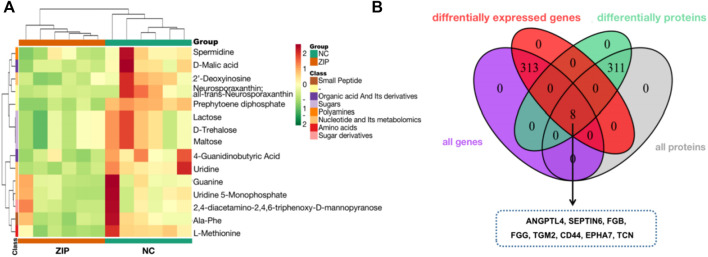
Metabolic analysis and Integrated analysis of SLC39A1-Overexpressed OSRC-2 cells. **(A)** Metabolites cluster analysis based on KEGG enrichment analysis result. **(B)** 8 genes have changes at both the transcriptional and protein levels.

Metabolomics data of RCC cell samples were analyzed to assess the metabolic difference. Principal Component analysis (PCA) and Orthogonal Partial Least Squares-Discriminant Analysis (OPLS-DA) demonstrated comprehensive metabolic change between two groups. The score plot of PCA (Figure 3A) and OPLS-DA (Figure 3B) witnessed significantly separated, indicating that SLC39A1 caused remarkable disturbance in RCC metabolism. Based on FC (Fold Change) ≥ 2 or ≤0.5 and VIP (Variable Importance in Projection) ≥ 1, 60 significant differential metabolites have been filtered out (Table 2). Functional annotations on differential metabolites by KEGG database was classified according to KEGG pathway types, and multiple metabolism variations were discovered: purine metabolism, pyrimidine metabolism, galactose metabolism, glutathione metabolism, and cAMP signal pathway (Figure 3C). MSEA enrichment analysis was able to prevent omissions of unapparent differentially expressed metabolites with important biological significance. The analysis demonstrated that SLC39A1 triggered alteration in more than 50 metabolism sets in MSEA (Figure 3D). In addition, the human metabolome database (HMDB) enrichment analysis revealed SLC39A1 also possessed noticeable impact on spermidine and spermine biosynthesis, lactose biosynthesis, GLUT-1 deficiency syndrome and congenital disorder of glycosylation CDG IId (Figure 3E). Then pathways involving no less than 5 differential metabolites were selected to perform cluster analysis. The heatmap displayed SLC39A1-regulated metabolites were clustered in nucleotides and its metabolomics, sugar and its derivatives, small peptide, and organic acid (Figure 4A).

**TABLE 2:** Identification results of significant differential metabolites in cell samples.

Metabolites	VIP	*p*_value	FC	Log_2_FC	Type
(R)-2-hydroxystearic acid	1.026262304	0.150466678	2.357333023	1.237155584	up
N-Oleoylethanolamine	1.794095424	0.003811691	0.382583326	−1.386154097	down
Uridine	1.862551429	0.003392615	0.44267699	−1.175673711	down
D-Trehalose	1.031047395	0.028538386	0.466447489	−1.100213419	down
Lactose	1.031047395	0.028538386	0.466447489	−1.100213419	down
Lactulose	1.031047395	0.028538386	0.466447489	−1.100213419	down
Maltose	1.031047395	0.028538386	0.466447489	−1.100213419	down
4-Guanidinobutyric Acid	1.644191608	0.006362563	0.478042103	−1.064790407	down
Phenyllactate (Pla)	1.174460381	0.022824344	0.484997939	−1.043949478	down
Uridine 5-Monophosphate	1.176790122	0.162335978	0.463132865	−1.110501956	down
N-lactoyl-phenylalanine	1.669737584	0.0098291	0.439787525	−1.185121413	down
D-Malic acid	1.627669732	0.018114668	0.495722017	−1.012396758	down
3-Methyluridine	1.929564294	0.002227522	0.319995889	−1.643874724	down
5-Hydroxy-2′-deoxyuridine	1.862551429	0.003392615	0.44267699	−1.175673711	down
Gly-Gly	1.571840697	0.002138695	0.430891368	−1.214603899	down
Adenosine 2′-Phosphate	1.316383154	0.152484703	0.41634288	−1.264155943	down
L-Methionine	1.776940312	0.00077552	0.492652152	−1.021358735	down
Asp-phe	1.046400676	0.358657024	0.495944425	−1.011749631	down
2′-Deoxyinosine	1.532960767	0.00715507	0.452891979	−1.142761107	down
2-Hydroxy-6-Aminopurine	1.155758566	0.241141732	0.465095063	−1.104402468	down
Oleamide	1.924806509	6.39059E-05	0.268566704	−1.896647638	down
Spermidine	1.396948582	0.025423149	0.420968131	−1.248217075	down
FFA (20:1)	1.727570459	0.002707229	0.265337637	−1.914098766	down
Gly-Phe	1.13556765	0.212600373	0.447247405	−1.160854984	down
N-(2-hydroxyethyl)stearamide	1.953727522	3.41233E-05	0.437311551	−1.193266638	down
2,4-diacetamino-2,4,6-triphenoxy-D-mannopyranose	1.071497895	0.281356736	0.492180041	−1.022741941	down
Guanine	1.155758566	0.241141732	0.465095063	−1.104402468	down
Ala-Phe	1.344414419	0.063840035	0.482970486	−1.049993067	down
Nicotinamide riboside (chloride)	1.309922868	0.063146704	0.333891167	−1.582550169	down
(E,Z)-2-Amino-3,14-octadecadien-1-ol	1.924806509	6.39059E-05	0.268566704	−1.896647638	down
Ile-Asp	1.010367737	0.40221139	0.496493079	−1.010154487	down
Bis(2-ethylhexyl) phthalate	1.887840954	0.000315942	0.456019919	−1.132831253	down
1-O-Hexadecyl-2-O-(2E-butenoyl)-sn-glyceryl-3-phosphocholine	1.498387225	0.015862398	0.497110428	−1.008361728	down
1-Oleoyl-2-acetyl-sn-glycerol	1.677918508	0.004816631	0.395767044	−1.337276614	down
5alpha-cholest-8-en-3-one	1.688626191	0.020456378	0.31539651	−1.664761398	down
Docosatrienoic acid	1.779951599	0.007774672	0.331983542	−1.590816375	down
Lagocholic acid	1.825654736	0.001145095	0.48324243	−1.049180963	down
Amastatin	1.319838137	0.460739062	0.439074016	−1.187463935	down
Cidofovir	1.089783659	0.351788917	0.478857045	−1.062333067	down
Macluraxanthone	1.709475948	0.014630029	0.402322996	−1.313573894	down
17-phenoxy trinor Prostaglandin F2 isopropyl ester	1.876280492	0.003048513	0.283684516	−1.817640687	down
Arg Tyr Ser	1.816846267	0.004637158	0.459449778	−1.122020924	down
Asp Ile Leu	1.238714749	0.253675276	0.455604991	−1.134144543	down
Asp-Ile	1.119726645	0.343004074	0.453756913	−1.140008473	down
Dihydro Isorescinnamine	1.889098004	0.001158257	0.263593609	−1.923612705	down
Glu Asn Ile Ile Asp	1.556418266	0.014100853	0.459065114	−1.123229293	down
Glu Glu Met Ile Ala	1.299664555	0.076952867	0.458315081	−1.125588337	down
Hexadecyl Acetyl Glycerol	1.881653456	1.20369E-05	0.425131424	−1.234019195	down
Huratoxin	1.659772823	0.000270397	0.470711454	−1.087085136	down
Lys Ile Val Lys	1.792448631	0.008780319	0.312597122	−1.677623601	down
N-dodecanoyl-L-Homoserine lactone-3-hydrazone-biotin	1.796933647	0.000749497	0.499898179	−1.000293823	down
Neomycin C	1.621473216	0.010855042	0.47575651	−1.071704698	down
Neurosporaxanthin;all-trans-Neurosporaxanthin	1.423578723	0.033931325	0.139068695	−2.846130392	down
Phe Lys Thr Glu	1.362690575	0.031184707	0.391329658	−1.353543642	down
Prephytoene diphosphate	1.930776567	1.06291E-06	0.418528927	−1.256600757	down
R-4-benzyl-3-((R)-3-hydroxy-2,2-dimethyloctanoyl)-5,5-dimethyloxazolidin-2-one	1.418030313	0.026251978	0.437099278	−1.193967099	down
Tyr Glu Gln Asp	1.815469014	4.49847E-05	0.464389266	−1.106593469	down
(6S,7R,8R,11S,12S,15R,16R)-7-(hydroxymethyl)-7,12,16-trimethyl-15-[(2R)-6-methyl-5-methylideneheptan-2-yl]pentacyclo [9.7.0.01,3.03,8.012,16]octadecan-6-ol	1.220343105	0.030324096	0.204957094	−2.286606167	down
[(2S)-2-pentadecanoyloxy-3-tetradecanoyloxypropyl] (4Z,7Z,10Z,13Z,16Z,19Z)-docosa-4,7,10,13,16,19-hexaenoate	1.008314167	0.009203128	0.378689054	−1.400914373	down
(2R,3R,4S,5S,6R)-2-[[(3S,4S,4aR,6aR,6bS,8aR,9R,12aS,14aR,14bR)-9-hydroxy-4-(hydroxymethyl)-4,6a,6b,8a,11,11,14b-heptamethyl-1,2,3,4a,5,6,7,8,9,10,12,12a,14,14a-tetradecahydropicen-3-yl]oxy]-6-(hydroxymethyl)oxane-3,4,5-triol	1.229648547	0.025360154	0.097710661	−3.355340208	down

### 3.4 Integrated proteomics, transcriptomics and metabolomics data

Transcriptomics and proteomics data described the relationship between proteins and genes. The Venn diagram illustrated that a total of 8 proteins were differentially expressed at both mRNA and protein levels. The heatmap showed the correspondence between regulation types of 8 proteins (Figure 4B).

Comprehensive analysis based on proteomics, transcriptomics and metabolomics data interpreted potential correlation between differentially expressed metabolites and genes. The Enrichment analysis was performed by mapping differential genes and metabolites to the KEGG pathway. SLC39A1 caused significant disturbances in metabolic pathways in renal cancer cells and altered transcriptional and translational levels of 71 genes (Supplementary Figure S4). The metabolism of several substances was remarkably affected: purine metabolism, pyrimidine metabolism, multiple amino acids metabolism, lactose metabolism, and free fatty acid metabolism. Combined data analysis showed that the increased abundance of SLC39A1 caused significant metabolic reprogramming.

**FIGURE 5:**
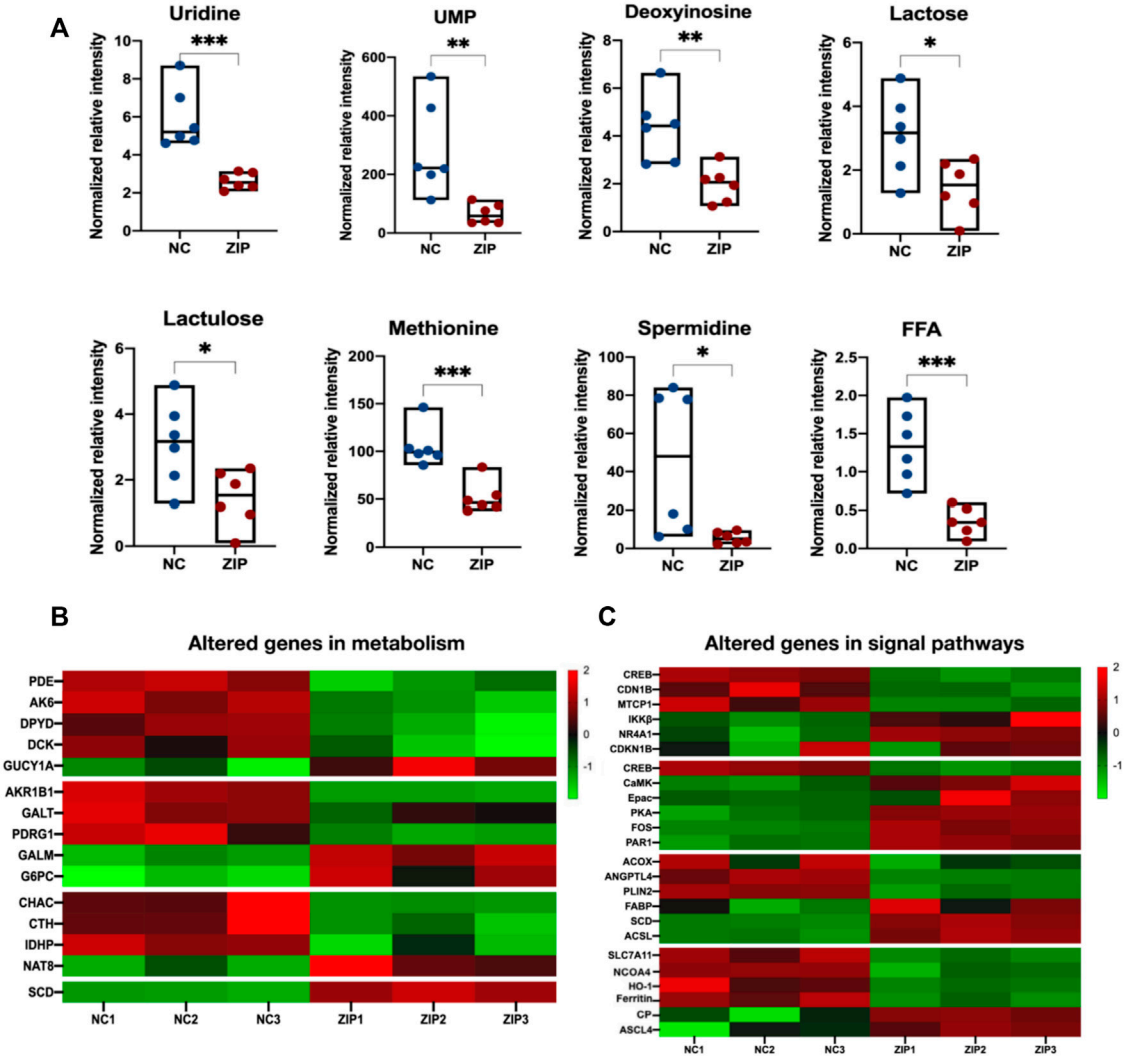
Integrated analysis result of altered metabolites and genes in metabolism and signal pathways. **(A)** 8 metabolites were indicated to be significantly altered and were all downregulated. The *Y*-axis displays the relative intensity and the *X*-axis the experimental groups. The upper and lower horizontal lines represent the maximum and minimum values, the middle lines represent the median, and the dots represent the samples. *p < 0.05, **p < 0.01, ***p < 0.001, as determined by Student’s *t*-test. **(B**,**C)** The heatmap of altered genes in metabolism and signal pathways triggered by SLC39A1 in OSRC-2 cells. Red indicates upregulated and green downregulated genes.

In nucleotide metabolism, significant downregulation of uridine, UMP, as well as upregulation of 2′-Deoxyinosine were observed (Figure 5A). Correspondingly, enzymes in pyrimidine and purine metabolism also changed: phosphodiesterase 2A (PDE2A), adenylate kinase 6 (AK6), dihydropyrimidine dehydrogenase (DPYD), Deoxycytidine kinase (DCK) expressions were decreased, and guanylate cyclase 1 soluble subunit alpha 1 (GUCY1A1/GCYA1) increased (Figure 5B). Galactose metabolism was also transformed, in which lactose and lactulose were reduced (Figure 5A). Aldoketo reductase family 1 member B (AKR1B1) and galactose-1-phosphate uridylyltransferase (GALT) were consumed, galactose mutarotase enzyme (GALM) and glucose-6-phosphatase (G6PC) were activated (Figure 5B). In amino acids and peptides metabolism, SLC39A1 reduced methionine in RCC cells and decreased cystathionine gamma-lyase (CTH) level (Figure 5A). SLC39A1 also participated in GSH metabolism, reducing spermidine production (Figure 5A). SLC39A1 downregulated CHAC1 (γ-GCTs, the glutathione-specific degradation enzymes) and isocitrate dehydrogenase-like protein (IDHP), while promoted N-acetyltransferase 8 (NAT8) (Figure 5B). In lipid metabolism, SLC39A1 significantly increased free fatty acids (Figure 5A), and stearoyl-CoA desaturase (SCD) expression level increased (Figure 5B).

Abnormal metabolic activities were generally accompanied with dysregulated oncogenic signal pathways (Park et al., 2020). SLC39A1 had been shown to regulate key genes in signal transduction pathways closely associated with tumorigenesis and development, involving PI3K-AKT signal pathway, cAMP signal pathway, PPAR signal pathway, and ferroptosis. In PI3K-AKT pathway, SLC39A1 reduced mature T cell proliferation 1 (MTCP1) and downregulated AKT downstream factor: cAMP-response element binding protein (CREB) and cyclin-dependent kinase inhibitor 1B (CDN1B), but up-regulated I-kappaB kinase beta (IKKβ). In cAMP signal pathway, calcium/calmodulin dependent protein kinase II gamma (CaMK) activity was activated. Exchange protein directly activated by cAMP (Epac) and PKA expression were upregulated. In PPAR signal pathway, fatty acid binding protein (FABP), SCD, and acyl-CoA synthetase long-chain (ACSL) were upregulated, with lower expression of angiopoietin like 4 (ANGPTL4), perilipin 2 (PLIN2), and acyl-CoA oxidase (ACOX). In addition, SLC39A1 also had important regulation on ferroptosis-related factors: solute carrier family 7 member 11 (SLC7A11), nuclear receptor coactivator 4 (NCOA4), heme oxygenase 1 (HO-1), and ferritin were decreased, and the expression of achaete-scute family bHLH transcription factor 4 (ASCL4) and ceruloplasmin (CP) were activated (Figure 5C).

**FIGURE 6:**
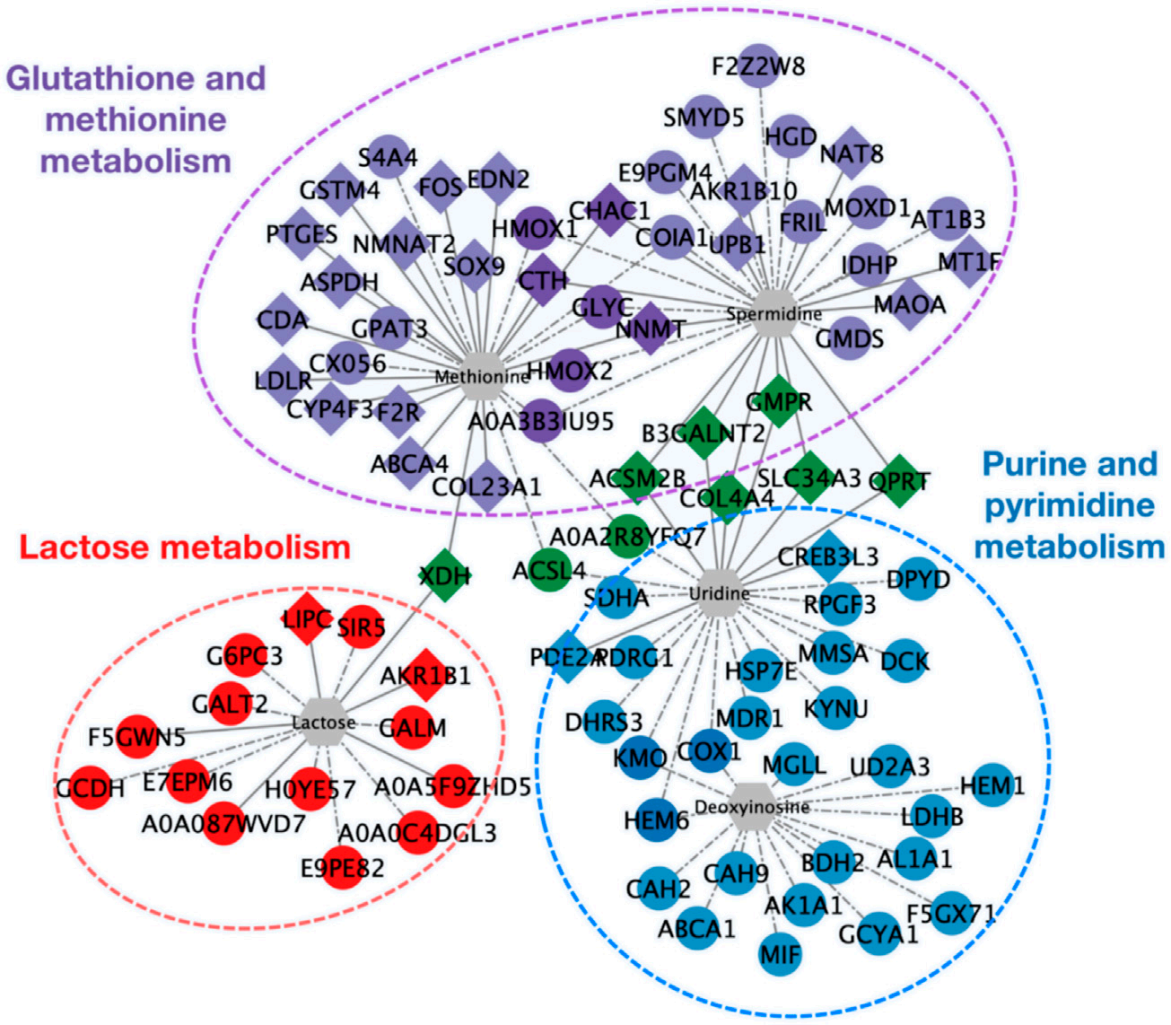
Network diagram of the interaction among differential expressed genes, proteins, and differential metabolites. Diamonds represent differential genes; circles represent differential proteins; hexagons represent differential metabolites. Realizations represent the interaction between differential expressed genes and differential metabolites; dashed lines represent the interaction between differential proteins and differential metabolites. Red indicates genes and proteins involved in lactose metabolism. Blue indicates purine and pyrimidine metabolism. Purple indicates glutathione and methionine metabolism. Green indicates differential expressed genes and proteins involved in the regulation of more than 2 types of metabolites.

To obtain a more intuitive demonstration of the association between differential genes, proteins, and differential metabolites, a correlation network was portrayed by Cytoscape (Figure 6). Differential genes and proteins caused significant disturbance in purine and pyrimidine metabolism, lactose metabolism, and glutathione and methionine metabolism. Changes in the mRNA or protein levels of enzymes (such as PDE2A, DPYD, DCK, AKR1B1, CHAC1, CTH, etc.) triggered alteration in several metabolites: uridine, deoxyinosine, spermidine, methionine, lactose, which was consistent with the results above.

## 4 Discussion

**FIGURE 7:**
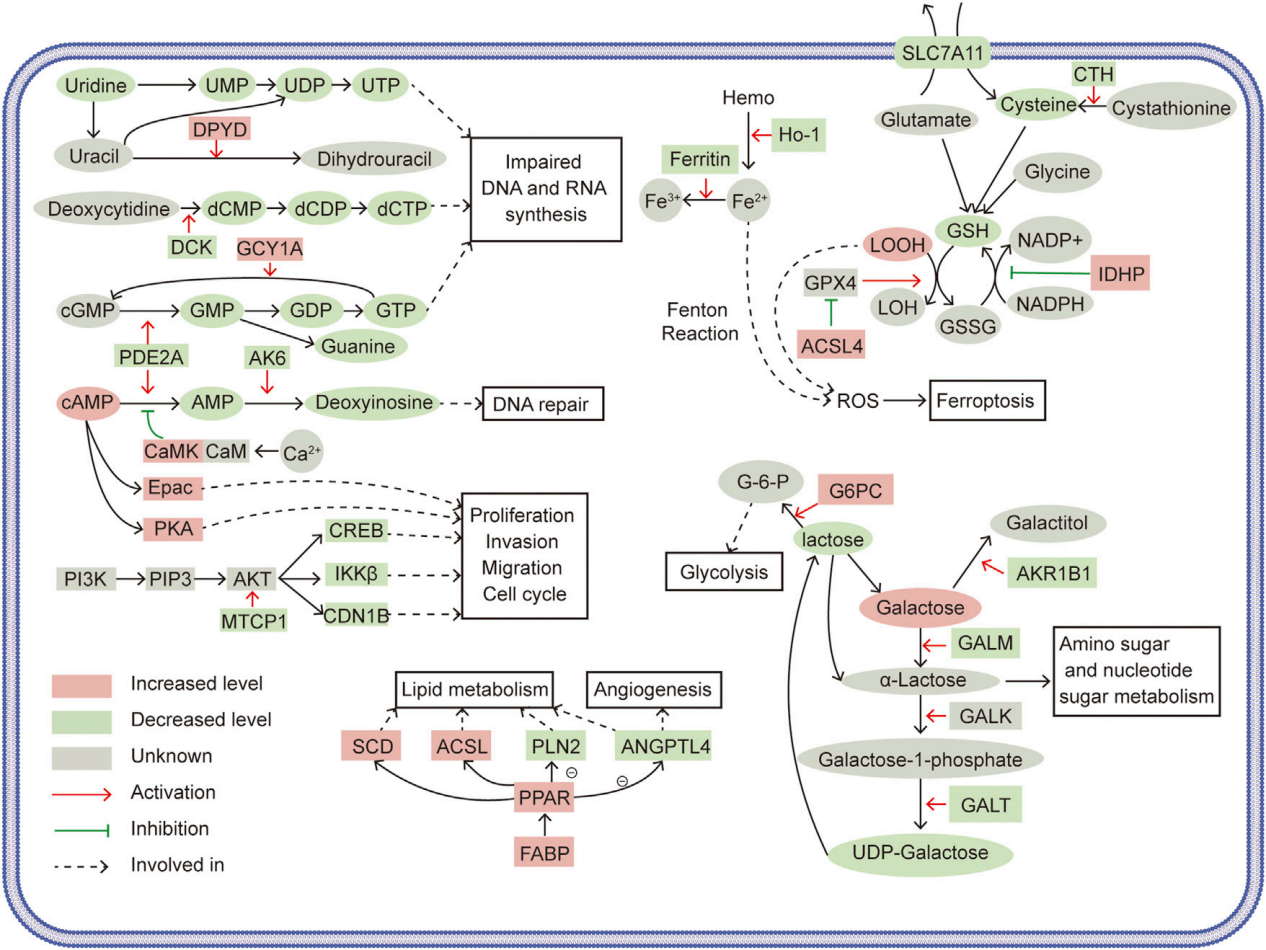
Schematics illustrating the tumor-suppressive mechanisms of SLC39A1. Ovals represent metabolites, squares represent related enzymes and regulatory proteins. Red indicates increased levels, green indicates decreased levels, and gray indicates temporarily unknown. Red arrows indicate activation, green arrows indicate inhibition, and black arrows indicate involved in.

Worldwide, RCC represented the seventh in estimated new cases and the eighth in estimated death, in all oncological occurrence (Siegel et al., 2021). SLC39A1 was uncovered to be low-expressed in a variety of tumors (Golovine et al., 2008; Desouki et al., 2015). Our previous studies found that the depletion of SLC39A1 promoted tumor proliferation and invasion in RCC (Dong et al., 2014). However, the underlying mechanism through which SLC39A1 inhibited tumor progression remained unclear. In this paper, we performed comprehensive analysis based on data form transcriptomics, proteomics and metabolomics and found significant several altered metabolism and signal transduction pathways in SLC39A1-overexpressed renal cancer cells. As shown in Figure 7, SLC39A1 caused significant alteration in metabolism and signaling pathways in RCC cells.

Unrestricted proliferation was the hallmark of biological function in tumor cells. Nucleotides are indispensable for cell proliferation and survival and are required for the biosynthesis of DNA and RNA, substance metabolism in cells (De Vitto et al., 2021). Purine metabolism and pyrimidine metabolism were perturbed in SLC39A1-overexpressed OSRC-2 cells. In purine metabolism, SLC39A1 reduced the synthesis of guanine and deoxyinosine. In terms of mechanism, SLC39A1 downregulated PDE2A to reduce the conversion of cGMP and cAMP into GMP and AMP and caused the upregulation of GCY1A to transform more GTP into cGMP. GMP was gradually converted to guanine in subsequent enzymatic reaction. Besides, AK6, in charge of converting AMP to deoxyinosine, was also significantly downregulated. And downregulation of uridine, UMP, and dCTP was observed in pyrimidine metabolism. SLC39A1 deregulated DPYD to promote the conversion of uracil to dihydrouracil, which increased the consumption of uracil. Dihydrouracil was gradually turned into β-alanine. The downregulation of DCK decreased the conversion of deoxycytidine into dCMP, leading to a decrease in dCTP level. In brief, we deduced that SLC39A1 impaired DNA and RNA production in RCC. SLC39A1 inhibited PDE2A to decrease UMP and subsequent UTP production. Downregulated DPYD and DCK increased the consumption of uracil and then diminished the production of dCMP, thereby reducing UTP, GTP and dCTP production. Deoxyinosine was also declined with augmented SLC39A1 and was reported to participate in DNA damage repair (Biswas et al., 2013).

Amino acid Metabolism was also altered: SLC39A1 decreased methionine, spermidine in renal cancer cell. CTH was downregulated by SLC39A1 and thus less cystathionine was turned into cysteine, resulting in the less interconversion between cysteine and methionine. Besides, SLC39A1 impeded GSH metabolism. The degradation of GSH starts with its hydrolysis to cysteinyl-glycine and oxoproline by CHAC1. After, cysteinylglycine was decomposed to produce cysteine and glycine. 5-Oxoprolinase generated glutamate *via* ATP hydrolysis (Traverso et al., 2013). NADPH acted as a hydrogen donor and promoted the transformation of GSSG into GSH, maintaining the reduce form of GSH (Kirsch and De Groot, 2001). SLC39A1 promoted the expression of IDHP, which converted NADP^+^ to NADPH, which might drive the interconversion between GSSG and GSH. Here, we found SLC39A1 reduced cysteine production by inhibiting CTH, impeding *de novo* synthesis of GSH, and upregulated CHAC1 to fuel the degradation of GSH.

In the classic Leloir pathway, galactose converted into UDP-glucose through enzymatic reactions. This four-step reactions were respectively mediated by GALM, galactokinase (GALK), GALT and UDP-galactose4-epimerase (GALE) (Demirbas et al., 2018). SLC39A1 induced significant decrease in lactose in RCC cells. Mechanistically, the downregulation of AKR1B1 brought about decrease the conversion of galactose into galactitol, raising the accumulation of galactose. The activation of GALM triggered lifted α-lactose production, which boosted amino sugar and nucleotide sugar metabolism. Downregulated GALT limited the production of UDP-galactose, consequently reducing lactose derived from UDP-galactose. In addition, SLC39A1 increased lactose consumption by upregulating G6PC to increase the conversion of lactose into glucose-6-phosphate (G-6-P), which was proverbially involved in glycolysis.

PI3K-AKT signal pathway, a classic cancer driver, was often abnormally activated in renal cell carcinoma (Guo et al., 2015). We observed that SLC39A1 inhibited PI3K-AKT signal pathway. The decreased MTCP1 suppressed the activation of AKT and AKT downstream factors, such as CREB, CDN1B and IKKβ, were also reduced. CREB was a transcription factor that largely high-expressed in RCC tissues. And CREB knock-down displayed inhibition on tumor proliferation *in vitro* and on tumor xenograft formation *in vivo* (Zhuang et al., 2016; Friedrich et al., 2020). Activated PI3K/AKT/CREB signal can promote cancer cell tumor proliferation, invasion, and cell cycle progression in pancreatic and prostate cancers (Tao et al., 2020; Meng et al., 2021). IKKβ was known as the key transcription activator of NF-κB and upregulated IKKβ protein expression is correlated to higher nuclear grade and significantly shorter survival (Krazinski et al., 2019). In cervical cancer, the activation of NF-κB by PI3K/AKT/IKKβ signal promoted the EMT process (Zhang et al., 2020). Besides, SLC39A1 downregulated GALT and knocking down GALT simultaneously deregulates multiple players in PI3K-AKT signal pathway (Tang et al., 2016). The cAMP signal pathway is another process significantly disturbed by SLC39A1. Besides, we speculated that cAMP signal was likely to be enhanced. Because SLC39A1 reduced conversion of cAMP into AMP by deregulating PDE2A, and upregulated CaMK to increase its phosphorylation inhibition on this conversion. Correspondingly, enhanced Epac and PKA levels, two commonly recognized cAMP receptors (Cheng et al., 2008), were also observed. Epac has contradictory effects in different cancer types and its function in renal carcinoma is rarely known (Wehbe et al., 2020). Taken together, our data suggested that SLC39A1 may suppress tumor proliferation, migration, and cell cycle by interfering PI3K/AKT and cAMP/Epac pathway in RCC cells.

Another significant altered pathway was PPAR signal pathway. We first noticed the high expression of FABP. FABP could bind to a variety of PPARs to stimulate PPAR signal pathway (Li et al., 2020). SLC39A1 upregulated FABP, which might allow more PPAR signal flowing into RCC cells. SLC39A1 showed differential regulation on PPAR targeted enzymes involved in lipid metabolism: SCD and ACSL were upward regulated, which might drive lipogenesis and fatty acid transport and ACOX was downregulated. Also, ANGPTL4 and PLIN2 that took part in adipocyte differentiation were deregulated (Monsalve et al., 2013). The transcriptional regulation of ANGPTL4 and its resulting expression could be determined by several transcription factors, including PPARα, PPARγ, PPAR-β/δ (La Paglia et al., 2017). Renal cell carcinoma was often characterized by hypoxia and HIF-drived increased tumor angiogenesis, glycolysis, and aberrant lipid metabolism (Inoue et al., 2014). In addition to HIF-1α, however, ANGPTL4 had also been confirmed as a conventional hypoxia-driven proangiogenic factor in renal cancer cells and this process could induced by PPAR signal pathway (Le Jan et al., 2003). Although ANGPTL4 was differentially expressed in both mRNA and protein levels in integrated analysis result and was also likely to participate in lipid metabolism (Monsalve et al., 2013), our present data was not able to prove this linkage. Overall, we suggested that tumor angiogenesis in renal cancer cell could be inhibited by SLC39A1 through downregulating ANGPTL4, and inactivated PPAR signal pathway was the underlying mechanism.

Ferroptosis referred to an iron-dependent cell death mode induced by the accumulation of intracellular lipid peroxides, which was remarkably different from classical apoptosis and necrosis in mechanism and implications. (Hirschhorn and Stockwell, 2019). The linkage between GSH and ferroptosis was inseparable: ferroptosis was mainly caused by lipid hydroperoxides overload in cellular membrane. GSH reduced lipid hydrogen peroxides to lipid alcohol by glutathione peroxidase 4 (GPX4), thereby suppressing ferroptosis (Koppula et al., 2021). Our data showed that SLC39A1 targeted serial key factors in ferroptosis. SLC39A1 downregulated SLC7A11 and raised ACSL4 expression. SLC7A11 could transfer extracellular cysteine into cytoplasm to facilitate glutathione synthesis and promoted the anti-lipid peroxidation effect of GPX4 (Stockwell et al., 2017; Koppula et al., 2021). ASCL4 was considered as a sensitive monitor as well as an important contributor of ferroptosis and eliminated the inhibition of GPX4 on ferroptosis in osteosarcoma cells, and overexpression of ACSL4 in glioma significantly reduced GPX4 level and contributes to ferroptosis (Yuan et al., 2016; Cheng et al., 2020). We also found that ferritin and HO-1 were downregulated by SLC39A1. Ferritin served as major storage of intracellular iron (Hou et al., 2016). The declining expression of ferritin upregulated intracellular iron, which triggered oxidative injury and induced ferroptosis (Dowdle et al., 2014). HO-1 produced Fe^2+^ by decomposing heme and was therefore considered to be an accelerator of ferroptosis. In summary, we speculated that SLC39A1 inhibited glutathione anabolism and accelerated its decomposition, resulting in impaired the adaption to oxidative stress of RCC cells, and promoted ferroptosis by deregulating the GSH-GPX4 system and altering other key proteins (such as SLC7A11, ACSL4, HO-1, and ferritin), ultimately fostering apoptosis in RCC cells.

ROS had long been associated with cancer and an augmented in various types of tumors (Panieri and Santoro, 2016). Elevated ROS levels damage DNA, proteins, and lipids to foster tumorigenesis, but excessive accumulation of ROS induces apoptosis in cancer cells (Liou and Storz, 2010). The antioxidant defense system in tumor cells was generally activated to scavenge superfluous ROS to maintain a certain level of ROS that promoted tumorigenesis and disease progress and meanwhile obtained apoptosis resistance (Gorrini et al., 2013). And GSH was regarded as the most important non-enzymatic antioxidant and therefore, utilized to attenuate oxidative stress (Kennedy et al., 2020). Recent study also demonstrated all types of RCC possess reduced oxidative phosphorylation capacity and dysregulated respiratory chain leading to electron leakage and excessive ROS. The level of GSH in RCC was tremendously increased to counteract the surge of ROS (Xiao and Meierhofer, 2019). Zn^2+^ could prevent cells from metabolic syndrome-associated oxidative stress (Olechnowicz et al., 2018b). SLC39A1 was essential for maintaining intracellular Zn^2+^ level and our data suggested that GSH biosynthesis was impeded by SLC39A1 in RCC cells. Thus, SLC39A1 possibly allowed RCC cells to poorer compatibility with high oxidative stress through regulating Zn^2+^ level and suppressing GSH abundance, the underlying mechanism of which required further research.

In addition, our study has some limitations. Our integrated analysis identified 8 proteins differentially expressed at both mRNA and protein levels, but these 8 proteins were not involved in SLC39A1-induced metabolic changes in RCC cells, which needed further exploration. Only one RCC cell line was involved in our research, and the findings of omics analysis and the specific role of Zn^2+^ in RCC needed to be proven in future experiments.

## 5 Conclusion

The present study illustrated that SLC39A1 functioned as a tumor suppressor in RCC. Integrated transcriptomics, proteomics and metabolomics data indicated that the anti-tumor effect might be related to altered purine and pyrimidine metabolism, glutathione metabolism and ferroptosis, generation of ROS, intervened PI3K-AKT, cCAMP-Epac and PPAR signal pathways. However, whether Zn^2+^ was an indispensable intermediary in the process of SLC39A1 inhibiting tumor progression remained to be explored. Our work drew a comprehensive blueprint for the underlying mechanism through which SLC39A1 suppresses renal cell carcinoma progression and provided novel insight into the development of therapeutic targets and potential biomarkers for this disease.

## Data Availability

The transcriptomic data have been submitted to the GEO repository and are accessible at the link (https://www.ncbi.nlm.nih.gov/geo/query/acc.cgi?acc=GSE208678). The proteomics data have been deposited to the ProteomeXchange Consortium (http://proteomecentral.proteomexchange.org) *via* the iProX partner repository with the dataset identifier PXD035469.

## Glossary

ACOX: acyl-CoA oxidase
ACSL: Acyl-CoA synthetase long-chain
AK6: adenylate kinase 6
AKR1B1: aldoketo reductase family 1 member B
ANGPTL4: angiopoietin like 4
ASCL4: achaete-scute family bHLH transcription factor 4
CaMK: calcium/calmodulin dependent protein kinase II gamma
CDN1B: cyclin-dependent kinase inhibitor 1B
CHAC1 γ-GCTs: the glutathione-specific degradation enzymes
CP: ceruloplasmin
CREB: cAMP-response element binding protein
C5AR1: completion C5a receiver 1
CTH: cystathionine gamma-lyase
DPYD: dihydropyrimidine dehydrogenase
Epac: Exchange protein directly activated by cAMP
ER: endoplasmic reticulum
FA: fatty acid
FABP: fatty acid binding protein
G-6-P: glucose-6-phosphate
G6PC: glucose-6-phosphatase
GALE: UDP-galactose4-epimerase
GALK: galactokinase
GALM: galactose mutarotase enzyme
GALT: galactose-1-phosphate uridylyltransferase
GPX4: glutathione peroxidase 4
GSH: glutamine
GSSG: glutathione disulfide
GUCY1A/GCYA: guanylate cyclase 1 soluble subunit alpha 1
HO-1: heme oxygenase 1
IDHP: isocitrate dehydrogenase-like protein
IKKβ: I-kappaB kinase beta
MTCP1: mature T cell proliferation 1
NAT8: N-acetyltransferase 8
NCOA4: nuclear receptor coactivator 4
PDE2A: phosphodiesterase 2A
PLIN2: perilipin 2
PPAR: peroxisome proliferators-activated receptor
RCC: Renal cell carcinoma
ROS: reactive oxygen species
SCD: stearoyl-CoA desaturase
SLC39A1: solute carrier family 39 member 1
SLC7A11: solute carrier family 7 member 11
TCA: cycle tricarboxylic acid cycle
