# Supplementary material for: Integrated analysis of transcriptomics, proteomics and metabolomics data reveals the role of SLC39A1 in renal cell carcinoma
Source: Front Cell Dev Biol. 2022 Nov 3;10:977960. doi: 10.3389/fcell.2022.977960 (PMC9669761; doi:10.3389/fcell.2022.977960)
Supplement: Supplementary file 1 [file DataSheet1.docx]

Supplementary Material

## Supplementary Figures


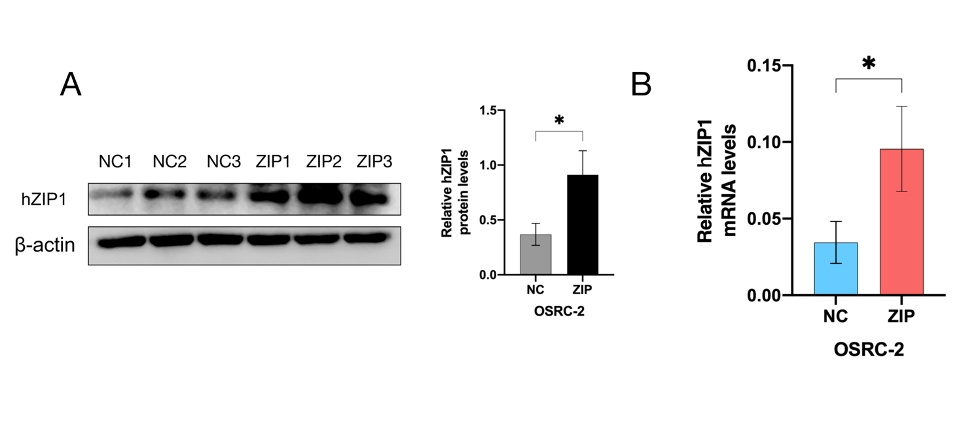


**Supplementary Figure 1. (A)** SLC39A1 protein level was measured in negative control and SLC39A1 overexpressed OSRC-2 cells (NC and ZIP) by western blotting analysis. **(B)** SLC39A1 mRNA level was measured in negative control and SLC39A1 overexpressed OSRC-2 cells (NC and ZIP) by real-time PCR analysis.


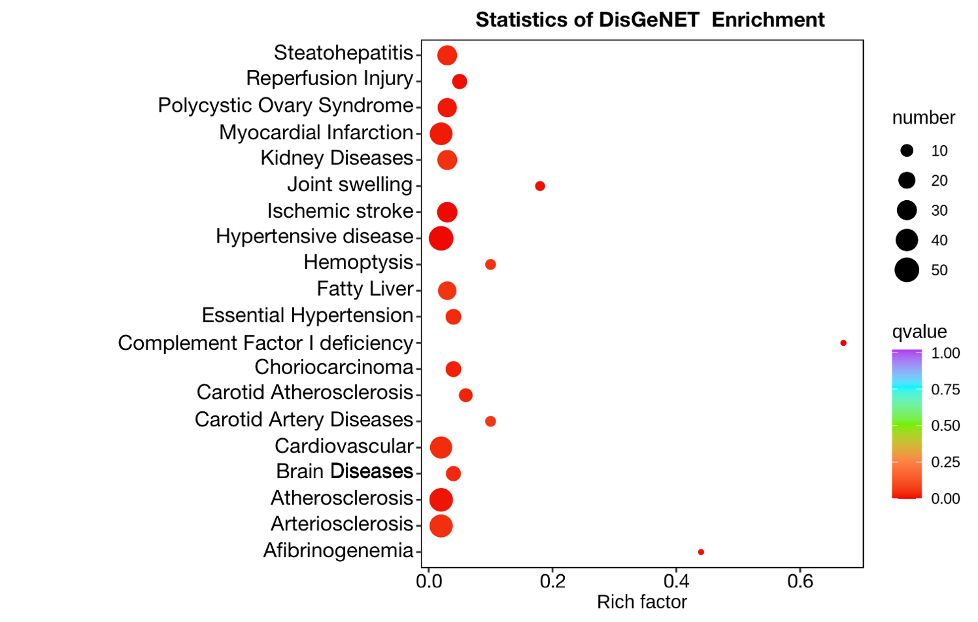


**Supplementary Figure 2.** DisGeNET enrichment analysis of transcriptomic data.


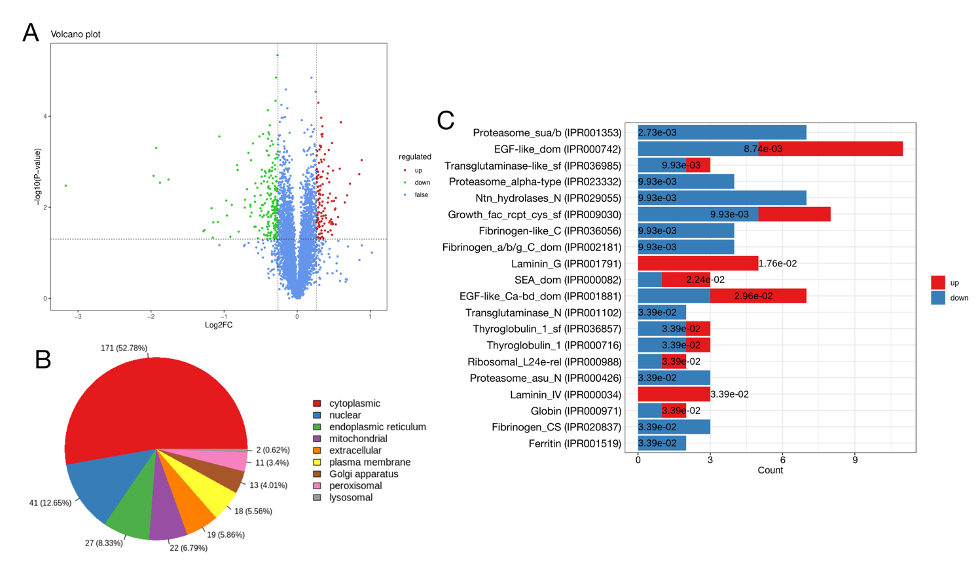


**Supplementary Figure 3. (A)** Differential proteins in SLC39A1 over-expressed OSRC-2 cells. **(B)** Subcellular localization of differential proteins. **(C)** Domain enrichment result of differential proteins.


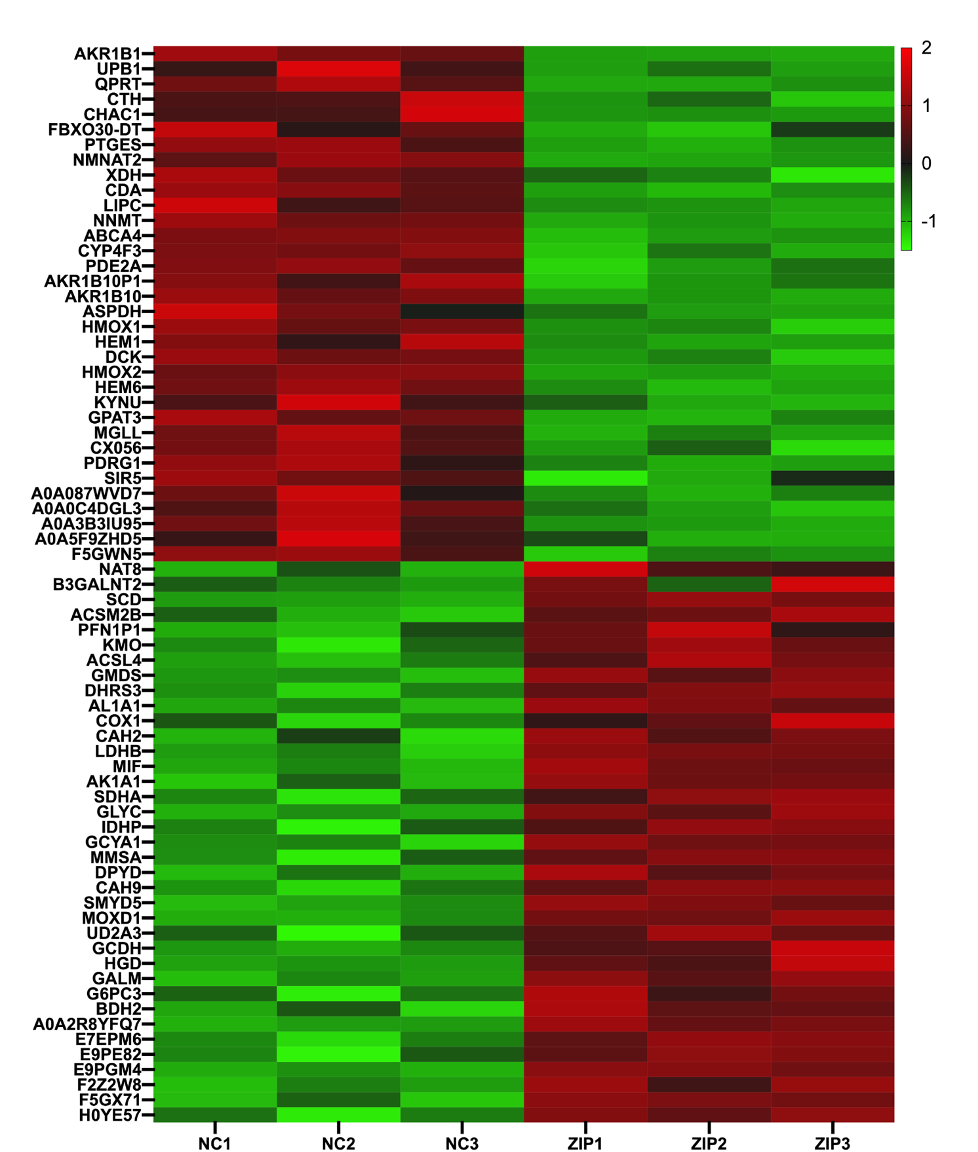


**Supplementary Figure 4.** Altered genes in metabolic pathways caused by SLC39A1.

**Supplementary Table**

**Supplementary Table 1.** Interactions of SLC39A1

| \| **Products** \| **Interactant** \| **Other Gene** \| **Complex** \| **Source** \| **Pubs** \| **Description** \| \| --- \| --- \| --- \| --- \| --- \| --- \| --- \| \| Q9NY26 \| Q14722 \| KCNAB1 \|  \| HPRD \| PubMed \|  \| \| Q9NY26 \| Q13303 \| KCNAB2 \|  \| HPRD \| PubMed \|  \| \| Q9NY26 \| Pleckstrin homology domain containing, family M (with RUN domain) member 2 \| PLEKHM2 \|  \| HPRD \| PubMed \|  \| \| Q9NY26 \| Q05513 \| PRKCZ \|  \| HPRD \| PubMed \|  \| \| Q9NY26 \| Q15464 \| SHB \|  \| HPRD \| PubMed \|  \| \| Q9NY26 \| Q9NY26 \| SLC39A1 \|  \| HPRD \| PubMed \|  \| \| Q9NY26 \| Q9NP94 \| SLC39A2 \|  \| HPRD \| PubMed \|  \| \| BioGRID:118050 \| BioGRID:128696 \| ADGRG5 \|  \| BioGRID \| PubMed \| Affinity Capture-MS \| \| BioGRID:118050 \| BioGRID:107180 \| C3AR1 \|  \| BioGRID \| PubMed \| Affinity Capture-MS \| \| BioGRID:118050 \| BioGRID:107189 \| C5AR1 \|  \| BioGRID \| PubMed \| Affinity Capture-MS \| \| BioGRID:118050 \| BioGRID:118992 \| CERS2 \|  \| BioGRID \| PubMed \| Two-hybrid \| \| BioGRID:118050 \| BioGRID:118886 \| CLEC2D \|  \| BioGRID \| PubMed \| Affinity Capture-MS \| \| BioGRID:118050 \| BioGRID:107707 \| COMT \|  \| BioGRID \| PubMed \| Two-hybrid \| \| BioGRID:118050 \| BioGRID:107875 \| CTLA4 \|  \| BioGRID \| PubMed \| Affinity Capture-MS \| \| BioGRID:118050 \| BioGRID:121805 \| CXCL16 \|  \| BioGRID \| PubMed \| Two-hybrid \| \| BioGRID:118050 \| BioGRID:110429 \| CXCL9 \|  \| BioGRID \| PubMed \| Two-hybrid \| \| BioGRID:118050 \| BioGRID:107914 \| CYB561 \|  \| BioGRID \| PubMed \| Two-hybrid \| \| BioGRID:118050 \| BioGRID:108511 \| FCGRT \|  \| BioGRID \| PubMed \| Affinity Capture-MS \| \| BioGRID:118050 \| BioGRID:108641 \| FPR2 \|  \| BioGRID \| PubMed \| Affinity Capture-MS \| \| BioGRID:118050 \| BioGRID:116380 \| FZD10 \|  \| BioGRID \| PubMed \| Affinity Capture-MS \| \| BioGRID:118050 \| BioGRID:109428 \| HNRNPH1 \|  \| BioGRID \| PubMed \| Affinity Capture-RNA \| \| BioGRID:118050 \| BioGRID:109590 \| HTR2C \|  \| BioGRID \| PubMed \| Affinity Capture-MS; Two-hybrid \| \| BioGRID:118050 \| BioGRID:109592 \| HTR4 \|  \| BioGRID \| PubMed \| Two-hybrid \| \| BioGRID:118050 \| BioGRID:108226 \| LPAR1 \|  \| BioGRID \| PubMed \| Affinity Capture-MS \| \| BioGRID:118050 \| BioGRID:120622 \| LSG1 \|  \| BioGRID \| PubMed \| Affinity Capture-MS \| \| BioGRID:118050 \| BioGRID:110484 \| MOV10 \|  \| BioGRID \| PubMed \| Affinity Capture-RNA \| \| BioGRID:118050 \| BioGRID:129568 \| NME2P1 \|  \| BioGRID \| PubMed \| Affinity Capture-MS \| \| BioGRID:118050 \| BioGRID:110916 \| NOTCH3 \|  \| BioGRID \| PubMed \| Reconstituted Complex \| \| BioGRID:118050 \| BioGRID:110925 \| NPC1 \|  \| BioGRID \| PubMed \| Affinity Capture-MS \| \| BioGRID:118050 \| BioGRID:115745 \| NXF1 \|  \| BioGRID \| PubMed \| Affinity Capture-RNA \| \| BioGRID:118050 \| BioGRID:122309 \| P2RY12 \|  \| BioGRID \| PubMed \| Affinity Capture-MS \| \| BioGRID:118050 \| BioGRID:111705 \| PTGER3 \|  \| BioGRID \| PubMed \| Affinity Capture-MS \| \| BioGRID:118050 \| BioGRID:117121 \| RIGI \|  \| BioGRID \| PubMed \| Affinity Capture-RNA \| \| BioGRID:118050 \| BioGRID:119539 \| SCARA3 \|  \| BioGRID \| PubMed \| Affinity Capture-MS \| \| BioGRID:118050 \| BioGRID:112432 \| SLC7A1 \|  \| BioGRID \| PubMed \| Affinity Capture-MS \| \| BioGRID:118050 \| BioGRID:115475 \| SORBS3 \|  \| BioGRID \| PubMed \| Affinity Capture-MS \| \| BioGRID:118050 \| BioGRID:121255 \| SPPL2B \|  \| BioGRID \| PubMed \| Affinity Capture-MS \| \| BioGRID:118050 \| BioGRID:112698 \| SUPT6H \|  \| BioGRID \| PubMed \| Affinity Capture-MS \| \| BioGRID:118050 \| BioGRID:125103 \| SYAP1 \|  \| BioGRID \| PubMed \| Affinity Capture-MS \| \| BioGRID:118050 \| BioGRID:112986 \| TNFRSF1A \|  \| BioGRID \| PubMed \| Affinity Capture-MS \| \| BioGRID:118050 \| BioGRID:130417 \| TRARG1 \|  \| BioGRID \| PubMed \| Two-hybrid \| \| BioGRID:118050 \| BioGRID:113275 \| VIPR2 \|  \| BioGRID \| PubMed \| Affinity Capture-MS \| \| BioGRID:118050 \| BioGRID:130382 \| YIPF6 \|  \| BioGRID \| PubMed \|  \| |
| --- | --- | --- | --- | --- | --- | --- | --- | --- | --- | --- | --- | --- | --- | --- | --- | --- | --- | --- | --- | --- | --- | --- | --- | --- | --- | --- | --- | --- | --- | --- | --- | --- | --- | --- | --- | --- | --- | --- | --- | --- | --- | --- | --- | --- | --- | --- | --- | --- | --- | --- | --- | --- | --- | --- | --- | --- | --- | --- | --- | --- | --- | --- | --- | --- | --- | --- | --- | --- | --- | --- | --- | --- | --- | --- | --- | --- | --- | --- | --- | --- | --- | --- | --- | --- | --- | --- | --- | --- | --- | --- | --- | --- | --- | --- | --- | --- | --- | --- | --- | --- | --- | --- | --- | --- | --- | --- | --- | --- | --- | --- | --- | --- | --- | --- | --- | --- | --- | --- | --- | --- | --- | --- | --- | --- | --- | --- | --- | --- | --- | --- | --- | --- | --- | --- | --- | --- | --- | --- | --- | --- | --- | --- | --- | --- | --- | --- | --- | --- | --- | --- | --- | --- | --- | --- | --- | --- | --- | --- | --- | --- | --- | --- | --- | --- | --- | --- | --- | --- | --- | --- | --- | --- | --- | --- | --- | --- | --- | --- | --- | --- | --- | --- | --- | --- | --- | --- | --- | --- | --- | --- | --- | --- | --- | --- | --- | --- | --- | --- | --- | --- | --- | --- | --- | --- | --- | --- | --- | --- | --- | --- | --- | --- | --- | --- | --- | --- | --- | --- | --- | --- | --- | --- | --- | --- | --- | --- | --- | --- | --- | --- | --- | --- | --- | --- | --- | --- | --- | --- | --- | --- | --- | --- | --- | --- | --- | --- | --- | --- | --- | --- | --- | --- | --- | --- | --- | --- | --- | --- | --- | --- | --- | --- | --- | --- | --- | --- | --- | --- | --- | --- | --- | --- | --- | --- | --- | --- | --- | --- | --- | --- | --- | --- | --- | --- | --- | --- | --- | --- | --- | --- | --- | --- | --- | --- | --- | --- | --- | --- | --- | --- | --- | --- | --- | --- | --- | --- | --- | --- |
